# Geography and Similarity of Regional Cuisines in China

**DOI:** 10.1371/journal.pone.0079161

**Published:** 2013-11-18

**Authors:** Yu-Xiao Zhu, Junming Huang, Zi-Ke Zhang, Qian-Ming Zhang, Tao Zhou, Yong-Yeol Ahn

**Affiliations:** 1 Web Sciences Center, School of Computer Science and Engineering, University of Electronic Science and Technology of China, Chengdu, People's Republic of China; 2 School of Informatics and Computing, Indiana University, Bloomington, United States of America; 3 Beijing Computational Science Research Center, Beijing, People's Republic of China; 4 Institute of Computing Technology, Chinese Academy of Sciences, Beijing, People's Republic of China; 5 Institute of Information Economy, Alibaba Business College, Hangzhou Normal University, Hangzhou, People's Republic of China; Umeå University, Sweden

## Abstract

Food occupies a central position in every culture and it is therefore of great interest to understand the evolution of food culture. The advent of the World Wide Web and online recipe repositories have begun to provide unprecedented opportunities for data-driven, quantitative study of food culture. Here we harness an online database documenting recipes from various Chinese regional cuisines and investigate the similarity of regional cuisines in terms of geography and climate. We find that geographical proximity, rather than climate proximity, is a crucial factor that determines the similarity of regional cuisines. We develop a model of regional cuisine evolution that provides helpful clues for understanding the evolution of cuisines and cultures.

## Introduction

The most essential need for all living organisms is energy, which is usually obtained by consuming food. So it is not hard to imagine why food affects all aspects of human life and culture [1]–[3]. Food has been studied in depth by many disciplines including history [4]–[7], sociology [8]–[10], philosophy [11], [12], literary criticism [13], etc. Understanding how food culture evolves will have profound impact on numerous domains. Despite its manifest importance, few studies have taken quantitative, systematic approaches towards food culture, mainly due to the scarcity of systematically collected databases.

Nevertheless, such an approach is promising. For example, one pioneering study revealed the connections between climate and the use of spices through the manual digitization of a large number of traditional recipes [14]. Recent increases in online recipe repositories have begun to allow for easier access to structured recipe data [15]–[17]. Harnessing this opportunity, we address the following questions about the evolution of regional cuisines: (1) How does the similarity between regional cuisines scale with geographical distance? (2) Is climate similarity the main factor determining similarity? Climate obviously plays an important role in shaping food culture, because it both limits the availability of ingredients and affects the usage of spices [14]. However, geographical proximity alone might drive nearby cuisines close because of frequent communication and migration. To address these questions, we examine regional recipes in China – the second largest and the most populous country in the world, which is home to over 1.3 billions of people with diverse cultural heritages. The landmass of China spans North-South and East-West rather evenly, providing a good test bed to study the effect of geography and climate.

Our investigation suggests that geographical distance alone plays a more important role than climate. Based on our results, we propose a model of cuisine evolution based on the copy-mutate mechanism. The key idea is that nearby regional cuisines tend to learn from each other either due to communication or migration. We demonstrate that our simple model reproduces important characteristics of the real data.

## Data and Methods

### Data collection

**Figure 1:**
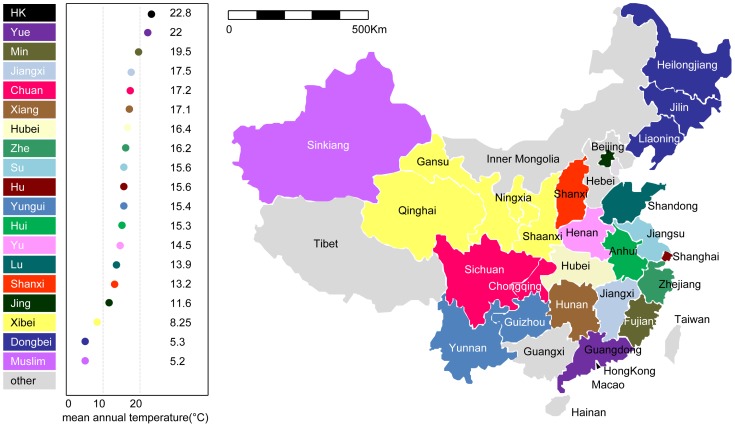
Map of regional cuisines in China.

**Figure 2:**
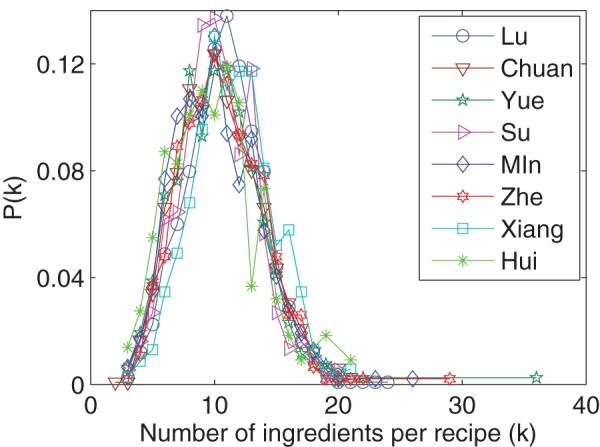
Probability distribution of the number of ingredients per recipe. All regional cuisines show similar distributions, which have a peak around 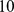.

In April 2012 we downloaded all the recipes from the Chinese recipe website *Meishijie* (http://www.meishij.net/), which categorizes all recipes into 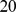 regional cuisines. Figure 1 shows a map of China and the mean annual temperature of each region, annotated with the names of provinces and cuisines, where the colors represent cuisines. Multiple provinces may belong to the same cuisine. Each recipe has the following properties: (i) a cuisine (each recipe belongs to only one cuisine); (ii) a list of ingredients; and (iii) a cooking method. We manually consolidated synonymous ingredient names and removed the recipes that do not have a cooking method or have too few (less than three) ingredients. There are 8,498 recipes and 2,911 ingredients in the cleaned dataset. Basic statistics of the dataset are reported in Table 1. Figure 2 shows that the number of ingredients per recipe is similarly distributed across all regions (the mode is around 10).

**Table 1:** The basic statistics of regional cuisines in China.

Regional Cuisine	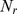	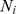	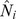	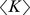	Location
**Lu**	1,066	788	64	10.8452	Shandong
**Chuan**	1,148	877	195	10.4608	Sichuan, Chongqing
**Yue**	775	900	23	10.2839	Guangdong
**Su**	372	573	88	10.3199	Jiangsu
**Min**	468	648	69	10.1880	Fujian
**Zhe**	460	594	14	10.5978	Zhejiang
**Xiang**	691	592	7	11.4660	Hunan
**Hui**	218	442	111	9.8899	Anhui
Dongbei	358	458	237	10.0363	Jilin, Heilongjiang, Liaoning
HK	151	367	210	9.4040	Hong Kong
Hubei	160	266	115	10.7375	Hubei
Hu	744	692	117	9.4274	Shanghai
Jiangxi	143	210	87	10.3147	Jiangxi
Jing	606	565	74	10.3614	Beijing
Other	52	171	16	9.5962	-
Muslim	521	426	131	10.7524	Sinkiang
Shanxi	125	191	5	11.4720	Shanxi
Xibei	188	338	52	10.8351	Shaanxi, Gansu, Qinghai, Ningxia
Yu	173	291	10	10.6936	Henan
Yungui	79	184	23	8.8101	Guizhou, Yunnan
**All**	8,498	2911	-	10.4399	-

There are 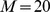 different regional cuisines in total. The eight major regional cuisines, labeled in bold, are the most representative and typical cuisines in China. 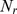: number of recipes. 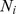: number of ingredients. 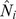: number of ingredients used only in the cuisine. 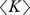: average number of ingredients in a recipe. The last column reports the provinces where a regional cuisine originates in.

**Figure 3:**
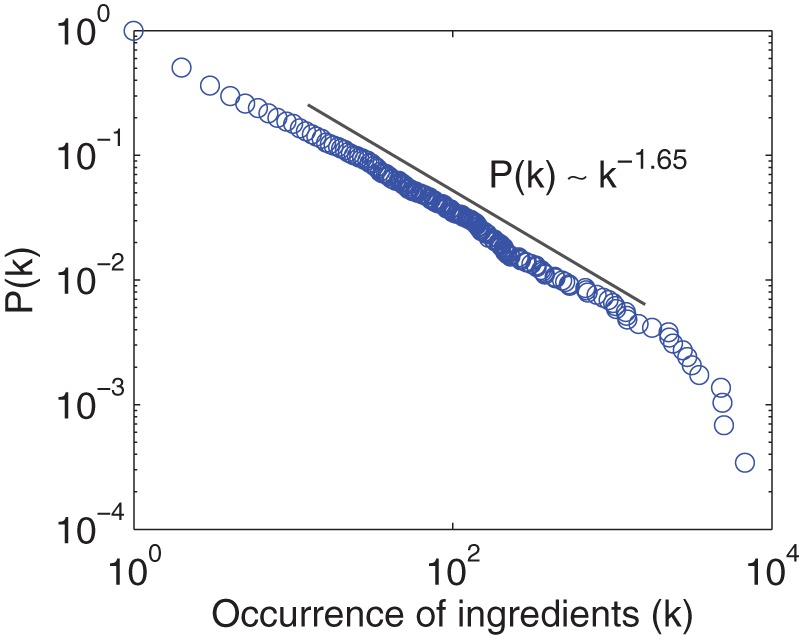
Cumulative frequency distribution of ingredient usage. The usage frequency is calculated using all recipes in our dataset. The exponent is obtained by the method in previous work [19].

**Figure 4:**
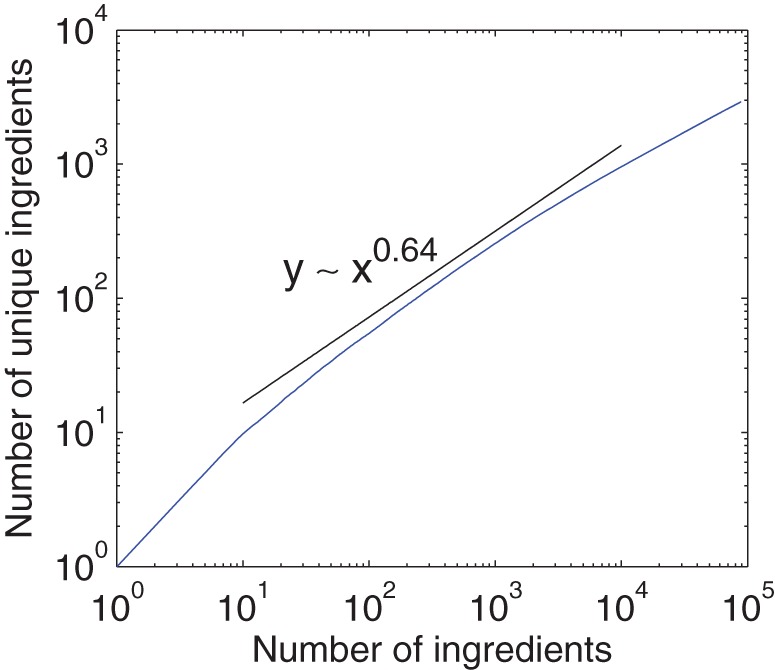
The number of distinct ingredients discovered vs. the number of recipes scanned. The plot (blue curve) approximately follows Heap's law (black guideline). The graph is obtained by averaging 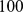 implementations with independently random sequences of recipes.

As previously observed, the ingredient usage frequency follows a skewed distribution; only a small fraction of popular ingredients are widely used, while many ingredients are observed in only a small number of recipes. As shown in Fig. 3, the frequency distribution of ingredients follows a power-law [18] (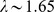 using method in paper [19]), capturing the intuition that a few ingredients such as salt, sugar, and egg constitute a major part of our every-day diet. As a result, the set of distinct ingredients roughly follows Heap's law, as seen in Fig. 4, with an exponent around 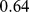. According to the method in previous work [20], the exponent of Zipf's law corresponding to Fig. 3 can be estimated by 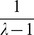. The product of this exponent and the exponent of Heap's law (0.64) is close to 1, which is consistent with the previous result [21].

### Quantifying similarity between cuisines

Our dataset can be considered as a bipartite network with a set of recipes and a set of ingredients. An edge between a recipe and an ingredient indicates that the recipe contains the corresponding ingredient. Since each recipe belongs to one and only one regional cuisine, the edges could be categorized into cuisines. Given a cuisine 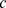 and an ingredient 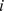, we use 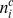 to denote the degree of ingredient 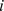, counted with edges in cuisine 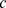. In other words, 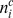 is the number of recipes (in cuisine 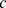) that use ingredient 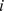. Therefore, the ingredient-usage vector of regional cuisine 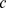 is written in the following form:
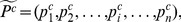
(1)where 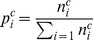 is the probability of ingredient 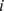 appears in cuisine 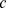. For example, if recipes in a regional cuisine 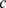 use 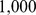 ingredients (with duplicates) in total and ingredient 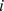 appears in 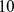 recipes in that cuisine, we have 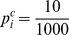.

Since common ingredients carry little information, we use an ingredient-usage vector inspired by TF-IDF (Term Frequency Inverse Document Frequency) [22]:
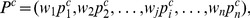
(2)where a prior weight 
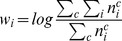
 is introduced to penalize a popular ingredient. We use 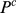 for all calculations in this paper. With this representation in hand, we quantify the similarity between two cuisines using the Pearson correlation coefficient (Eq. 3) and cosine similarity (Eq. 4).

- Pearson product-moment correlation [23]: This metric measures the extent to which a linear relationship is present between the two vectors. It is defined as
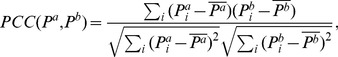
(3)where 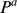 and 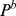 are ingredient-usage vectors of regional cuisine 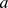 and 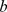, respectively.
- Cosine similarity [24]: It is a measure of similarity between two vectors of an inner product space that measures the cosine of the angle between them. For regional cuisines 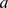 and 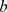, the cosine similarity is represented using a dot product and magnitude as
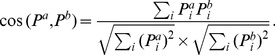
(4)

## Results

### Outlier detection

**Figure 5:**
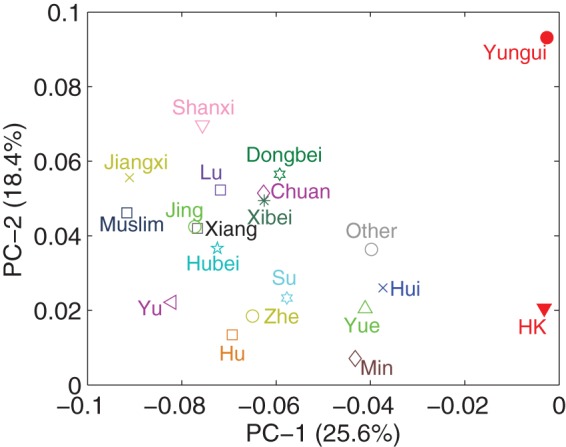
The distribution of regional cuisines in the first two principal component spaces. YunGui and Hong Kong (red) stand out as outliers.

As an overview, we apply Principal Component Analysis to identify principal components of the ingredient usage matrix [25]. The distributions of regional cuisines in two principal components (capturing 44% of the information) are presented in Fig. 5. The two most obvious outliers (in solid red) are Yungui and Hong Kong cuisine. This may reflect the facts that ethnic minorities have historically resided in the Yungui region and that Hong Kong was ruled by the British Empire and Japan for more than 100 years.

### Geography and climate

Climate shapes ingredient-usage patterns not only by affecting the availability of ingredients but also by exerting other pressures, such as a need to use additional spices as preservatives [14]. At the same time, we expect nearby regions to have a higher probability of similar food culture even without similar climate, because they are more likely to have more communication and migration.

To estimate the effect of climate we use temperature as a proxy. We assume that the annual average temperature approximately captures one of the most fundamental aspects of climates. As shown in the previous work on spices [14], annual temperature strongly predicts the usage of spices, and we further assume that temperature is a strong climate factor that affects ingredient availability. For two regions 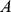 and 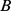, the temperature difference 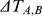 is simply 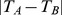, where 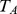 is the annual average temperature of region 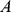.

**Figure 6:**
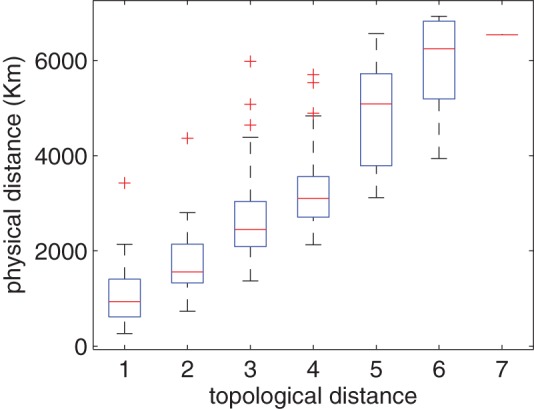
Topological distance vs. physical distance.

We quantify geographical proximity using two distance measures: physical distance and topological distance. We measure physical distance between two cuisines by identifying the central cities of the cuisines and then calculating the great-circle distance [26]. To measure topological distance between two cuisines, we construct a graph of cuisines, where a node represents a regional cuisine and an edge represents the adjacency of two cuisines, we then measure topological distance by the shortest path length on the graph. Figure 6 shows that the geographical distance and topological distance are correlated yet exhibit large variance.

**Figure 7:**
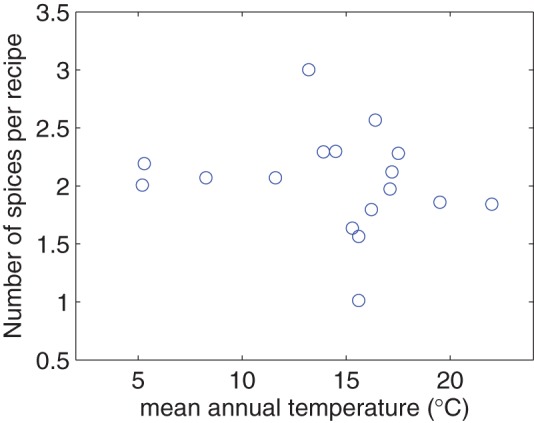
Number of spices per recipe vs. mean annual temperature.

Figure 7 shows the relationship between the number of spices per meat-based recipe in a region and the mean annual temperature of the region. The correlation is insignificant (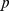-value is 0.238), in contrast to the results of the previous work [14]. Our result may arise due to the fact that China is still a single country with smaller temperature variation than the whole world.

**Figure 8:**
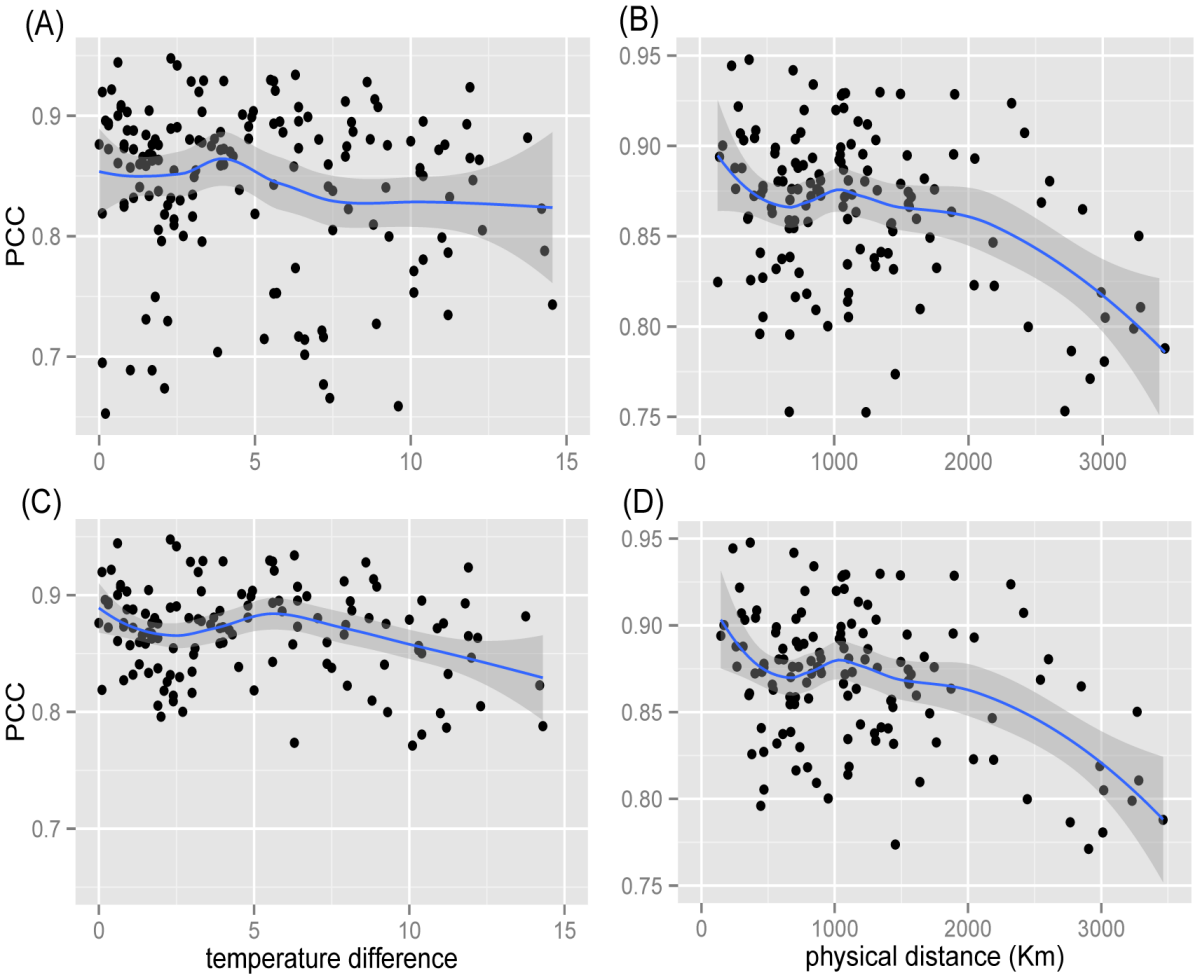
The dependence of similarities between different regional cuisines on the climate and geography. (A): scatter plot of PCC and temperature difference (all regional cuisine pairs); (B): scatter plot of PCC and physical distance (all regional cuisine pairs); (C): scatter plot of PCC and temperature difference (neglecting outliers); (D): scatter plot of PCC and temperature difference (neglecting outliers).

Figure 8 compares how temperature and physical distance are related to the similarities between regional cuisines. The left column shows the results of temperature and the right column shows that of physical distance. Each circle represents a pair of cuisines. The Pearson correlation coefficient between the temperature difference and PCC is −0.134 (Figure 8A), indicating a weak correlation between similarity of regional cuisines and their temperature difference. When we delete the two outliers mentioned above (Yungui and HK), the Pearson correlation coefficient between the temperature difference and PCC becomes −0.216 (Figure 8C). That is, regions with similar temperature tend to share similar usage patterns of ingredients, which is consistent with previous results [14]. However, this may not be the effect of temperature, because climate is correlated with distance. The Pearson correlation coefficient between the physical distance and PCC is −0.289 (Figure 8B), indicating a stronger correlation. When neglecting outliers, it becomes −0.385 (Figure 8D). The 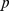-values of all cases indicate significant difference (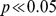 for both cases).

The previous analysis does not provide a complete picture, since geography and climate are strongly correlated; nearby regions are more likely to have similar climates. To estimate the effect of climate and geographical proximity, we calculate partial correlation [27], which is used to measure the linear association between two factors while removing the effect of other additional factors. The partial correlation coefficients between physical distance and PCC, given temperature difference as a control variable, is −0.280. However, the partial correlation between temperature difference and ingredient usage similarity, given physical distance as a control variable, the expected negative correlation completely vanishes and the correlation coefficient becomes 0.116. Our results indicate that the effect of temperature on the ingredient usage pattern may not exist at all. The results with cosine and the cases without outliers also show the same tendency.

**Figure 9:**
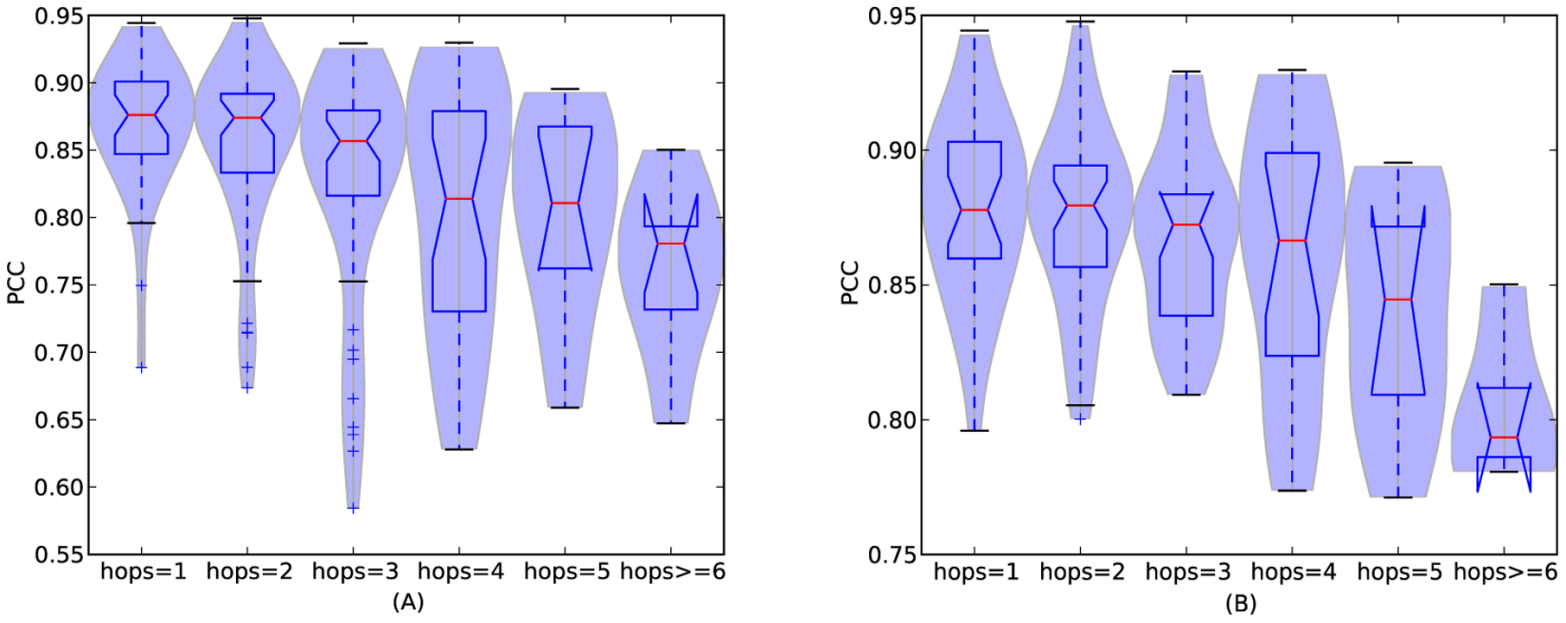
Similarity distributions of regional cuisine pairs with different topological distance (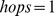, 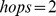, 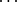, 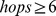). (A): result for all regional cuisines; (B): result when neglecting outliers.

Here we examine the relationship between topological distance and the similarity of cuisines. Figure 9 shows the similarity distribution of cuisines with respect to topological distance. Analysis of variance (ANOVA) [28] shows that the difference in the similarity distribution is significant (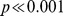 for both cases). The figure shows a clear trend that geographically closer regional cuisines have more similar ingredient usage patterns. We perform a simple permutation significance test by classifying all regional cuisine pairs into two classes: the first class contains regional cuisine pairs with topological distance less than or equal to 2, and the second class contains those of topological distance larger than 2. Denote the similarities of two classes as 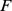 (far, with topological distance 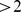) and 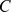 (close, with topological distance 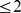), and 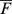 and 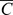 is the mean of 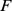 and 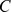, respectively. 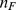 and 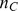 are the sample sizes corresponding to each group. The null hypothesis 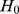 says the two classes 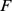 and 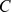 have identical probability distributions. We performed the test as follows. First, the difference between 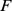 and 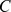 is calculated. This is the observed value of the test statistic, namely 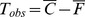. The observations of classes 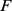 and 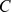 are then pooled. Next, the difference in sample means is calculated and recorded for every possible way of dividing these pooled values into two groups of size 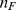 and 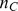, denoted by 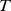. Lastly, the one-sided 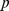-value of the test is calculated as 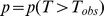. The 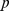-values of Fig. 9 indicate significant difference (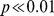 for both cases).

## Model and Validation

We build an evolution model of Chinese cuisines based on the simple notion that geographical proximity breeds more communication and migration. Our model uses the copy-and-mutate model of recipe evolution [16]. We tested two models that use topological distance and physical distance, respectively, and found that the model using topological distance produces better results. There are three elements in the model: regional cuisines, recipes, and ingredients. We assume the same set of Chinese regional cuisines in the same location. A recipe is a set of ingredients and belongs to one of the regional cuisines. We assume that each recipe has exactly the same number of ingredients (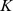). Each ingredient 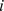 has a fitness value 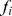, randomly drawn from a uniform distribution in 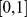. This fitness represents intrinsic properties such as nutritional value, flavor, cost, and availability [16]. All the symbols used in our model are listed in Table 2.

**Table 2:** Notations for parameters and quantities in the model.

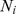	number of all ingredients
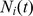	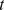 number of initial ingredients at time
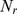	number of all recipes
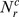	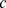 number of recipes in the regional cuisine
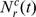	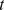 number of initial recipes in the regional cuisine 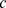 at time
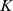	number of ingredients per recipe
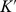	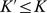 number of ingredients to be mutated in each recipe,
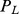	probability of interaction
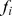	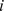 the fitness of ingredient
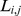	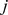 the topological distance of regional cuisine 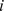 and

Let us describe our model in detail. In the initial state there are 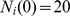 ingredients in the ingredient pool, and each regional cuisine contains one recipe that consists of 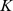 random ingredients chosen from the initial ingredient set; that is, 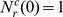.

### Step 1

Choose one regional cuisine preferentially. Regional cuisine 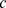 is chosen with probability
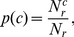
(5)where 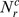 is the number of recipes of regional cuisine 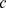 and 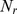 is the total number of recipes of all regional cuisines. With probability 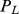 the chosen regional cuisine (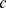) will interact with another region (**Step 2**). With probability 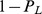 cuisine 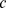 will develop a recipe itself by randomly selecting 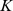 unique ingredients from the ingredient pool.

### Step 2

Generate a recipe by learning from others. With probability proportional to their topological distance, cuisine 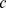 will select another regional cuisine 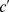. Regional cuisine 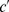 will be chosen with probability
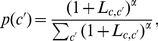
(6)where 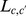 is the topological distance between cuisine 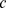 and 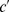. 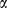 is a free parameter to tune the importance of topological distance. Regional cuisines with smaller topological distance from cuisine 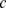 will have higher probability to be chosen in this step when 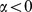. Assuming cuisine 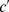 is chosen to interact with 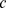, then cuisine 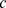 will randomly choose one recipe of cuisine 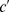 as a template and copy it. From this copy, we randomly chose an ingredient 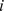 and compare it with an ingredient 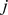 that is randomly chosen from the ingredients pool, if 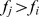, we replace ingredient 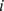 by ingredient 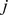. This process is repeated 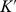 times. We then execute **Step 3**.

### Step 3

Add new ingredients. With probability 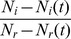 we add one new ingredient to the ingredients pool and replace the ingredient having the smallest fitness in the recipe that was generated in the previous steps with the new ingredient in order to assure all the ingredients in the ingredient pool have been used. We then add the modified new recipe to the pool of cuisine 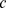. If no new ingredient is added in this step, we then add the new recipe to the pool of cuisine 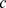 without modification. If we already have 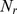 recipes, stop the simulation, or else repeat the previous steps.

**Figure 10:**
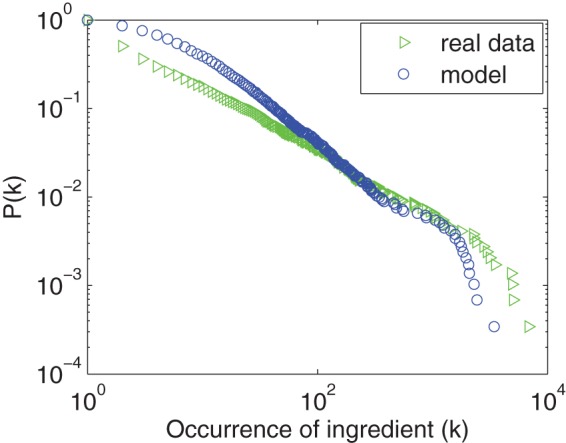
Cumulative frequency distribution of the cuisine network generated by our model compared to the the empirical distribution from the dataset of Chinese cuisine.

The parameters 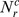 and 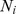 are obtained from the data. For simplicity, we set 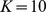, which means that every recipe has ten unique ingredients. In the initial state, there are 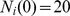 ingredients in the ingredient pool, and each regional cuisine contains one recipe chosen randomly from the initial ingredients; that is, 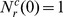. The cuisines then evolve as new recipes and ingredients join over time. We used 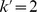, 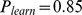, and 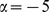, which generates results that closely resemble the empirical findings. Figure 10 demonstrates that our model produces a qualitatively similar, skewed degree distribution.

**Figure 11:**
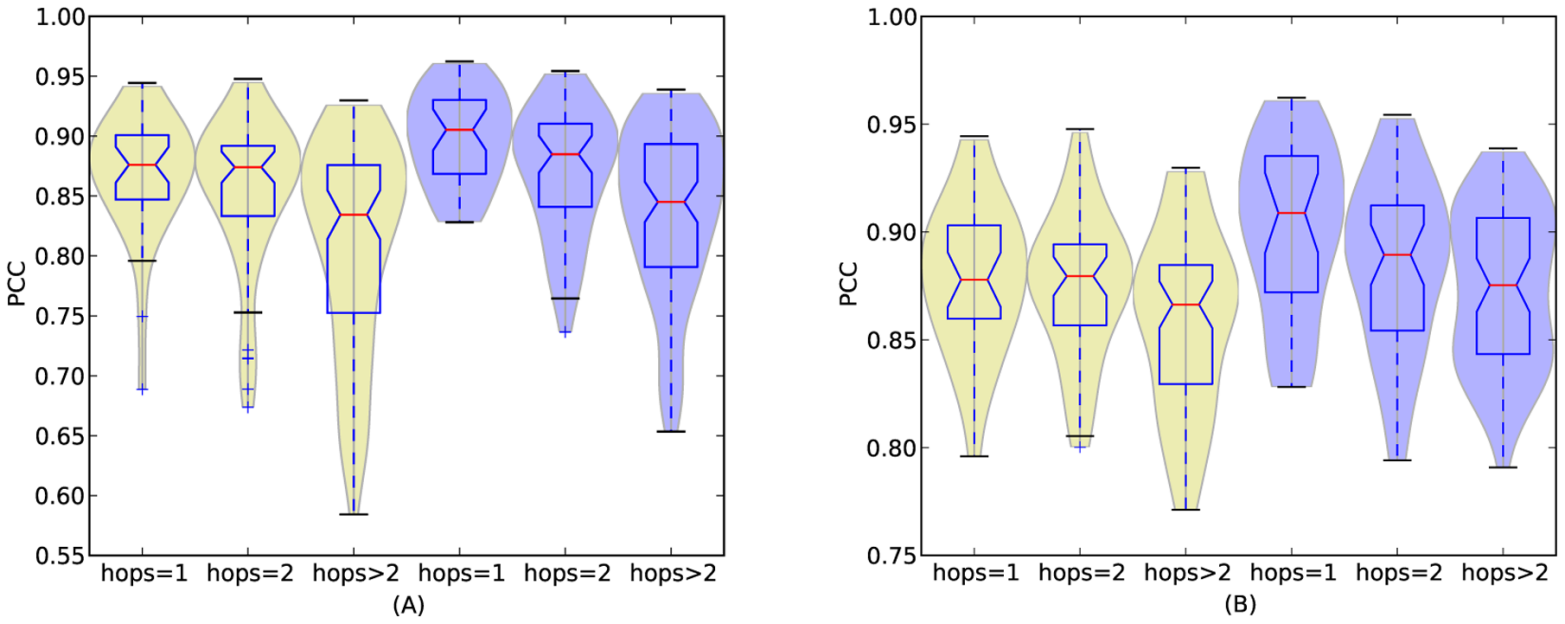
Similarity distributions of regional cuisine pairs with different topological distance (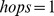, 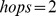 and 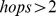). The blue ones are the results generated by our model, while the yellow ones are results of the dataset. (A): result for all regional cuisines; (B): result when neglecting outliers.

**Figure 12:**
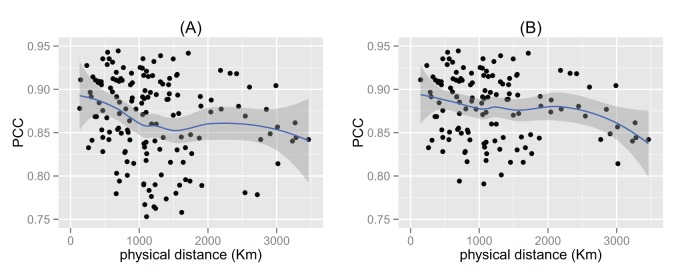
The dependence of similarities between different regional cuisines on the geographical distance in the model. (A): result for all regional cuisines; (B): result when neglecting outliers.

Figure 11 shows the comparison between the real data and our model in terms of the similarity distribution with topological distance. Fig. 11A is the result of all regional cuisine pairs, displaying that our model can achieve the similar tendency as the real dataset, although the real dataset shows more diversity than our model. We think this is a result of the existence of outliers in the dataset. Fig. 11B shows the result without outliers, displaying a better match with our dataset. The liner correlation between the results of the model and the dataset are both larger than 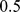, indicating a strong correlation. Figure 12 shows the dependency between physical distance and cuisine similarity in our model. The results display a similar tendency found in the real data (B and D in Fig. 8). Results of cosine similarity are not displayed, as all of them display similar tendencies with PCC.

## Conclusion and Discussion

We empirically analyzed the similarity relations between major regional cuisines in China in terms of climate and physical proximity. We found that climate (temperature) does not show any correlation with ingredient usage similarity if we control geographical distance, while geographical proximity seems to be a key factor in the shaping of regional cuisines. Based on the finding, we proposed a copy-and-mutate model that incorporates geographical proximity. We showed that the results of our model agree with our empirical findings.
